# Willingness to Be Contacted via a Patient Portal for Health Screening, Research Recruitment, and at-Home Self-Test Kits for Health Monitoring: Pilot Quantitative Survey

**DOI:** 10.2196/59837

**Published:** 2024-11-22

**Authors:** Elizabeth Lockhart, Jordan Gootee, Leah Copeland, DeAnne Turner

**Affiliations:** 1 Public Health Sciences, Michigan State University + Henry Ford Health, 1 Ford Place, Suite 5E, Detroit, MI, 48202, United States, 1 3137997237; 2 College of Nursing, University of South Florida, Tampa, FL, United States

**Keywords:** patient portals, patient engagement, personal health records, risk assessments, health information, information access, open notes, user perceptions

## Abstract

**Background:**

Patient portals are being increasingly used by health systems in the United States. Although some patients use portals for clinical use, patient perspectives on using portals for research-related activities, to complete health screenings, and to request at-home self-test kits are unclear.

**Objective:**

We aimed to understand patient perspectives on using electronic health portals for research; health-related screenings; and patient-initiated, home-based self-testing.

**Methods:**

Patients (N=105) from the Patient Engaged Research Center at a large, urban, midwestern health system completed a 23-item web-based survey on patient portal (MyChart) use and willingness to use the patient portal for research, risk assessments, and self-test kits. Frequencies and percentages were generated.

**Results:**

Almost all participants (102/105, 97.1%) had accessed MyChart at least once, with most (44/102, 43.1%) indicating they logged in at least once per month. Participants indicated logging into MyChart to check laboratory results or other health data (89/105, 84.8%), because they received a message to log in (85/105, 81%), and to message their health care professional (83/105, 79%). Fewer participants logged in to see what medications they had been prescribed (16/105, 15.2%) and to learn more about their health conditions (29/105, 27.6%). Most participants indicated logging into MyChart on a computer via a website (70/105, 66.7%) or on a smartphone via an app (54/105, 51.4%). When asked about how likely they would be to participate in different types of research if contacted via MyChart, most (90/105, 85.7%) said they would be likely to answer a survey, fill out a health assessment (87/105, 82.9%), or watch a video (86/105, 81.9%). Finally, participants would be willing to answer risk assessment questions on MyChart regarding sleep (74/101, 73.3%), stress (65/105, 61.9%), diabetes (60/105, 57.1%), anxiety (59/105, 56.2%), and depression (54/105, 51.4%) and would be interested in receiving an at-home self-test kit for COVID-19 (66/105, 62.9%), cholesterol (63/105, 60%), colon cancer (62/105, 59%), and allergies (56/105, 53.3%). There were no significant demographic differences for any results (all *P* values were >.05).

**Conclusions:**

Patient portals may be used for research recruitment; sending research-related information; and engaging patients to answer risk assessments, read about health information, and complete other clinical tasks. The lack of significant findings based on race and gender suggests that patient portals may be acceptable tools for recruiting research participants and conducting research. Allowing patients to request self-test kits and complete risk assessments in portals may help patients to take agency over their health care. Future research should examine if patient portal recruitment may help address persistent biases in clinical trial recruitment to increase enrollment of women and racial minority groups.

## Introduction

Patient portals are widely implemented throughout health care systems in the United States. As part of the 2014 Cures Act [1], these secure, web-based applications can give patients convenient access to their personal health information anytime they have access to an internet connection. In 2020, about 60% of individuals in the United States were offered access to a patient portal, and almost 40% accessed one [2]; however, national data regarding access and use of patient portals in the United States after the COVID-19 pandemic have not yet been released.

Patients use patient portals for a variety of clinical functions, including viewing laboratory results, requesting refills, messaging health care professionals, requesting appointments, and viewing educational materials related to their health care and self-management improvement [2,3]. Patients’ willingness to use portals may be influenced by their age, ethnicity, education level, health literacy, and health status [4,5]. Some barriers, such as concerns regarding confidentiality [6], preferring to speak directly with their physician [7,8], and technology access [3], also limit an individual’s engagement with patient portals.

Increasing the scope of functions within a patient portal may help facilitate patient access to health care, increase engagement, and allow patients to take agency over their health care. Despite their potential, portals have been underused for providing access to screenings, risk assessments, and patient-initiated self-test kits. Offering self-testing in portals provides increased access to health care for patients who may have transportation issues or fear entering a health facility in person for stigmatized conditions, such as HIV and sexually transmitted infections. In addition to clinical use, portals have the potential to be used to recruit patients for participation in research studies [9]. Patients have reported a preference for being contacted for research opportunities via electronic methods [10]. However, recruitment rates via patient portals vary; some studies reported recruiting fewer patients [11] when using patient portals versus other methods (eg, over the phone, in person, and via letters), while others were able to enroll more patients [12]. Additionally, the impact of patient portals on equitable recruitment is unclear. Research has found that White patients, when recruited via portals, are more likely to participate in research compared to Black, Hispanic, or Asian patients [13]. However, the increased engagement with patient portals among racial and ethnic minority patient groups following the onset of the COVID-19 pandemic [14] has the potential to increase exposure to recruitment and participation in research. The types of research that patients would participate in if approached via a patient portal are unclear; it is also unclear if patients would be willing to complete research tasks within the portal itself.

Patient portals offer a variety of benefits to patients, but opportunities remain to broaden their use. Patients would benefit from further access to these portals and possibly even broader utilization of what patient portals can offer, such as answering risk assessments and self-testing. Further research is needed to determine patient preferences for using portals and health conditions for which patients may be willing to use patient portals. This exploratory work examines how patients view patient portals for screening, risk assessments, self-testing, and research.

## Methods

### Study Population and Participant Recruitment

Participants were recruited from a convenience sample within the Patient Engaged Research Center (PERC) at Henry Ford Health to participate in a brief electronic survey. Any person with experience as a patient or caregiver could become a patient advisor within the PERC by completing an application, phone screen, and onboarding workshop. The role of a patient advisor includes, but is not limited to, providing feedback about ways to improve health care. A Qualtrics (Qualtrics International Inc) link for the survey was initially sent to all 406 patient advisors via email and a monthly electronic newsletter; 105 responded and completed the survey. Reminder emails were sent to patients approximately 2 weeks and 4 weeks after the initial email to remind those who had not yet completed the survey.

### Survey Instrument

A total of 23 questions were developed by the research team regarding the patient portal (MyChart; Epic Systems Corporation), including questions about using the patient portal for research purposes and for requesting self-test kits for health. Participants were asked to provide information on their race (Black, White, American Indian or Alaska Native, or Native Hawaiian or Pacific Islander), age, marital status (married, widowed, divorced, separated, or never married), and gender (male, female, nonbinary, another gender, or prefer not to say).

Responses to questions regarding reading information about health conditions on MyChart, answering screening questions on MyChart, and whether participants would likely participate in research if contacted via MyChart were measured on a 5-point Likert scale (extremely likely to extremely unlikely). Participants were asked if they had ever heard of preexposure prophylaxis (yes, no, or unsure) and which method of HIV testing they would prefer (at the doctor’s office, taking a home-based test on their own, or not wanting or needing an HIV test). Participants were also asked how they access MyChart, which health topics (diabetes, blood pressure, HIV, drug use, alcohol use, tobacco use, cancer, cholesterol, depression, stress, anxiety, physical activity, diet, sleep, asthma, use of seat belts, immunizations, and sexual history) they would be willing to answer questions about in MyChart, and which self-test kits (colon cancer, COVID-19, HIV, sexually transmitted infections, allergies, cholesterol, influenza, urinary tract infections, alcohol use, and illicit drug use) they would be interested in receiving if they were provided free of charge.

### Data Analysis

All data were exported, cleaned, and uploaded into SPSS (IBM Corp). Frequencies and descriptive statistics were generated. Chi-square tests were run to examine whether significant differences existed among demographic variables.

### Ethical Considerations

The PERC is covered by an overarching institutional review board (IRB) approval at Henry Ford Health (IRB#16766) for patient advisors to fill out surveys and provide preliminary feedback. This study was covered under a blanket IRB approval for research conducted among PERC patient advisors and was considered preparatory to research; participants did not need to sign a consent form. All study data were deidentified. Anyone who completed the survey and indicated interest was entered into a raffle for three US $75 gift cards.

## Results

### Participant Characteristics

Participants were on average 57 (SD 14.35) years old (Table 1). Most identified as White (69/105, 65.7%) and female (80/105, 76.2%). Over half were married (60/105, 57.1%). For all results presented, there were no significant differences in responses by gender or race (all *P* values were >.05). All participant characteristics can be found in Table 1.

**Table 1.** Participant characteristics (total sample: N=105).

Characteristics		Participants, n (%)
Gender		
	Male	23 (21.9)
	Female	80 (76.2)
	Nonbinary/other	2 (1.9)
Age (years)		
	18‐24	2 (1.9)
	25‐44	17 (16.2)
	45‐64	49 (46.7)
	≥65	37 (35.2)
Race and ethnicity		
	White	69 (65.7)
	Black	31 (29.5)
	Another race	5 (4.8)
Marital status		
	Married	60 (57.1)
	Widowed	6 (5.7)
	Divorced	18 (17.1)
	Never married	21 (20)
Ever accessed MyChart		102 (97.1)

### Patient Portal Usage

Almost all participants had accessed MyChart (102/105, 97.1%), with the majority accessing it at least once per month (44/102, 43.1%). There were various reasons why patients used MyChart, and patients reported various methods of accessing MyChart (Table 2).

**Table 2.** Frequency of logging into, reasons to log into, and methods of accessing MyChart.

		Participants, n (%)^a^
Frequency of MyChart log-in		
	Daily	6 (5.9)
	At least once a week	30 (29.4)
	At least once a month	44 (43.1)
	At least once within 6 months	17 (16.7)
	At least once a year	3 (2.9)
	Never	2 (2)
Reasons to log into MyChart		
	Because I received a message/text/email to log in	85 (81)
	To pay a bill	51 (48.6)
	To message my provider	83 (79)
	To schedule/reschedule an appointment	62 (59)
	To check laboratory results/other health data	89 (84.8)
	To refill medication	40 (38.1)
	To learn more about health conditions	29 (27.6)
	To see which medications I have been prescribed	16 (15.2)
Methods of accessing MyChart		
	On a smartphone via the app	54 (51.4)
	On a smartphone via the website	27 (25.7)
	On a computer via the website	70 (66.7)
	On a tablet via the app	13 (12.4)
	On a tablet via the website	10 (9.5)

a Participants could select more than 1 option or none. As such, n values and percentages do not add up to 105 and 100%, respectively, at all instances.

### Research via Patient Portal

Participants were likely to participate in different types of research if they were contacted by researchers in MyChart, including answering surveys, watching videos, and completing health assessments. They were also willing to answer questions to assess their risk for health conditions, including diabetes, HIV, and substance use (Table 3).

**Table 3.** Participants’ willingness to participate in research and risk assessments in MyChart and preferences for research recruitment.

		Participants, n (%)^a^
Somewhat or very likely to participate in research if contacted via MyChart		
	Answer a survey	90 (85.7)
	Watch a video	86 (81.9)
	Fill out a health assessment	87 (82.9)
	Research that involves a medical device	66 (62.9)
	To give biomedical samples (blood, saliva, hair sample)	72 (68.6)
Most favored way to hear about research studies		
	MyChart message	38 (38.8)
	Text message	12 (12.9)
	Telephone call	3 (3.3)
	Email	23 (24)
	Letter from provider	14 (14.7)
	Provider telling me about opportunities verbally at my appointment	16 (16.8)
Being willing to answer risk assessment questions on MyChart for the following conditions		
	Diabetes	60 (57.1)
	HIV	33 (31.4)
	Drug use	37 (35.2)
	Alcohol use	42 (40)
	Tobacco use	35 (33.3)
	Depression	54 (51.4)
	Stress	65 (61.9)
	Anxiety	59 (56.2)
	Sleep	74 (70.5)
	Sexual history	35 (33.3)

a Participants could select more than 1 option or none. As such, n values and percentages do not add up to 105 and 100%, respectively, at all instances.

### Self-Test Kits

When asked what self-test kits they would be willing to receive if free of charge, participants indicated interest in several health conditions (Table 4).

**Table 4.** Interest in receiving a self-test kit.

Test kit type	Participants, n (%)^a^
Colon cancer	62 (59)
COVID-19	66 (62.9)
HIV	17 (16.2)
Sexually transmitted infections	23 (21.9)
Allergies	56 (53.3)
Cholesterol	63 (60)
Influenza	51 (48.6)
Urinary tract infections	53 (50.5)
Alcohol use	13 (12.4)
Illicit drug use	13 (12.4)
None	6 (5.7)
All	27 (25.7)

a Participants could select more than 1 option or none. As such, n values and percentages do not add up to 105 and 100%, respectively.

## Discussion

### Principal Findings

Electronic health records and their patient portals, such as MyChart, have become increasingly common. We found that patients overwhelmingly use MyChart and are willing to use it to answer risk assessments, participate in research, watch videos, read health information, and complete surveys.

Importantly, we found no significant demographic differences in having used MyChart, accessing MyChart, completing activities within MyChart, and being willing to use MyChart for research purposes (all *P* values were >.05). The majority of our participants (38/98, 38.8%) were willing to not only be contacted to participate in research via the portal but also complete research-related activities, such as taking risk assessments (87/105, 82.9%) and viewing videos (86/105, 81.9%) in the portal. The lack of significant results in this study is in contrast to previous research. Other research has found differences in patient portal use based on demographic factors [15-17]. However, those studies were almost exclusively conducted before the COVID-19 pandemic. As Mai and colleagues [14] found, COVID-19 may be leading to a shrinking of the racial digital divide, as access and use of portal functionalities have expanded among racial minority groups. Patient portals may be tools for reaching large numbers of patients [18] for research purposes while potentially increasing access to research participation for women [19] and racial minority groups [14], who have historically not been equally represented in research. Future research should examine if research recruitment via patient portals can enroll participants who align with the demographic makeup of the health care system.

Participants in our research were generally willing to complete risk assessments via the patient portal and receive self-test kits, including those for stigmatized topics like HIV and substance use. In a similar fashion, a scoping meta-analysis by Gnambs and Kasper [20] found that individuals are more likely to disclose sensitive behaviors in computer-assisted surveys. Future research should examine whether answering questions about stigmatized topics in patient portals results in increased patient–health care professional communication or preventive care regarding those topics. Self-testing for HIV and substance use is important, as individuals who may not seek a health care professional for testing may be more comfortable or willing to test on their own [21,22]. As the TakeMeHome project found [23], providing HIV self-test kits gives options to people who may not be able or willing to test otherwise. Future research should examine if offering patients HIV self-test kits via the patient portal increases HIV testing in health care systems.

There are some limitations to this research. First, this study occurred with patients who were part of the PERC. Patients who participate in activities with the PERC may be different from the health care system patient population overall. For example, they are more likely to participate in research because they are already part of a research center and are more engaged with the health care system. Second, participants in this research were more likely to be female than male. Female individuals, compared to male individuals, have been found to be more likely to use patient portals [24]. The results may have been different if more male individuals participated. Finally, the survey for this research was distributed electronically. It may be that people who participate in web-based surveys are more likely to feel comfortable doing health-related activities electronically.

### Conclusions

Patient portals allow patients to both have access to their electronic health information and interact with the health care system. They are becoming increasingly more common for clinical communication, but as we found, they can also be used for research recruitment and sending research-related information. Patients generally prefer to hear about research studies via portals and are willing to use them to answer risk assessments, read about health information, and complete other clinical tasks. Furthermore, patients would be interested in receiving self-test kits for various health conditions, including those for sensitive health conditions such as HIV. Patient portals are relatively novel tools for engaging patients in a variety of research and clinical activities, and patient portal use may be an ideal way to engage patients in research related to sensitive health conditions.

## Abbreviations

IRB: institutional review board
PERC: Patient Engaged Research Center
